# A Feasibility Study of Multimodal Optical Coherence Tomography Angiography and Elastography for Assessing Foot Microvascular Changes and Elasticity Following Repeated Mechanical Loading

**DOI:** 10.3390/s26206631

**Published:** 2026-10-20

**Authors:** Yu Tang, Zhengshuyi Feng, Guangyu Zhang, Jiacheng Gu, Yilong Zhang, Zhihong Huang, Chunhui Li

**Affiliations:** 1 School of Physics, Engineering and Technology, University of York, York YO10 5DD, UK; fdr519@york.ac.uk (Y.T.); srx522@york.ac.uk (Z.F.); xgq516@york.ac.uk (J.G.);; 2 Centre for Medical Engineering and Technology (CMET), School of Science and Engineering, University of Dundee, Dundee DD1 4HN, UK

**Keywords:** OCTA, OCE, foot microcirculation, mechanical loading, foot elasticity

## Abstract

The foot is regularly exposed to mechanical loading during daily activities. Repeated loading can affect both microvascular and mechanical properties of foot tissue. Optical coherence tomography angiography (OCTA) and optical coherence elastography (OCE) provide non-invasive methods for assessing these changes. This feasibility study evaluated combined OCTA and OCE imaging for measuring vascular and mechanical responses in healthy foot skin following repeated loading in 10 healthy volunteers. The dorsal and plantar regions of the foot were imaged using a portable swept-source OCT system. Baseline measurements were acquired first, followed by three repeated loading cycles (C1, C2, and C3). For each cycle, the same force was applied for 30 s. The force differed between participants according to individual tolerance and averaged approximately 25 N across the experiment. OCTA and OCE acquisition began approximately 3 s after force release to observe changes in vascular and mechanical parameters following repeated loading. In the plantar region, vessel skeleton density (VSD) showed a significant overall difference between loading conditions (*p* = 0.006). Young’s modulus showed significant overall differences in both the dorsal and plantar regions (both *p* < 0.001). Relative to baseline, the dorsal Young’s modulus was 22.9%, 32.3%, and 39.0% higher following C1, C2, and C3, respectively. The corresponding values in the plantar region were 18.1%, 29.2%, and 39.0% higher. Repeated-measures ANOVA identified significant loading effects for VSD, vessel area density (VAD), and Young’s modulus, together with a significant region effect for VSD. These findings demonstrate the feasibility of combined OCTA and OCE imaging for assessing vascular and mechanical responses in healthy foot tissue following repeated loading. This method may support future studies of foot tissue response in populations with altered microcirculation or tissue mechanics, including people at risk of diabetic foot complications.

## 1. Introduction

The foot is continuously exposed to mechanical stress during daily activities. The plantar skin and subcutaneous tissues help absorb shock, redistribute pressure, and protect underlying structures [1]. Accumulated mechanical loading can change plantar skin blood flow [2]. In people with diabetes, microvascular dysfunction and altered tissue elasticity can impair the response to stress or injury [3,4]. Excessive plantar tissue stress is an important factor in diabetic foot ulcer development, especially in people with peripheral neuropathy [5]. Diabetic foot ulcers are a serious complication of diabetes and are associated with delayed healing, infection, amputation, and increased mortality [6,7,8,9]. Plantar soft tissue thickness and stiffness are important biomechanical properties involved in load bearing and pressure redistribution [10,11,12]. Therefore, assessment of both microvascular and mechanical responses to loading may provide useful information on foot tissue behavior.

Current clinical foot assessment methods include visual inspection, palpation, monofilament testing, Doppler ultrasound, and plantar pressure mapping [13]. However, these methods have several limitations. They can be subjective, lack quantitative stiffness measurement, have limited resolution for capillary imaging, and cannot assess subcutaneous mechanical properties [14,15]. A biopsy provides more detailed information but is invasive. Other imaging methods, such as magnetic resonance imaging (MRI) and computed tomography (CT), have limited resolution for microvascular and mechanical assessment [16,17,18]. Therefore, non-invasive and high-resolution imaging methods that can assess microvascular and mechanical properties of foot tissue are needed [19].

Optical coherence tomography (OCT) is a non-invasive imaging technique that enables high-resolution visualization of skin structure [20,21,22,23]. However, conventional OCT mainly provides morphological information and does not quantify microvascular or biomechanical properties [24]. To address this, functional extensions such as optical coherence tomography angiography (OCTA) and optical coherence elastography (OCE) have been developed. OCTA allows visualization of microvascular perfusion and provides vascular density and flow-related information [25,26]. OCE enables the assessment of tissue mechanical properties, including stiffness-related parameters such as Young’s modulus [27,28].

Previous studies have used OCTA to assess microvascular changes in the foot under baseline conditions and after external stimulation. One commonly used method is local heating. OCT was used to measure microvascular structure and blood flow in the foot under baseline conditions and after thermal stimulation [29]. Another approach is post-occlusive reactive hyperemia, in which temporary blood flow occlusion is induced using a pressure cuff, followed by OCTA imaging after cuff release to assess vascular recovery [30]. However, these approaches mainly focus on vascular responses and do not consider concurrent changes in tissue mechanics. Under repeated or sustained loading, local blood flow and tissue mechanical behavior change [2]. Chronic microvascular dysfunction and altered tissue mechanical properties may impair the response to mechanical stress or injury [3,10]. Excessive plantar tissue stress is also associated with diabetic foot ulcer development [5]. Assessing short-term vascular and mechanical responses to loading may, therefore, provide functional information on tissue response and vulnerability beyond baseline measurements. Characterizing these responses in healthy tissue may provide a reference for future studies in people with diabetes.

Multimodal OCTA–OCE provides a method for the combined assessment of vascular parameters and tissue elasticity. For the foot, such a combined assessment may be useful because repeated loading can affect both microvascular and mechanical responses [31,32]. In this study, the same marked skin sites on the dorsal and plantar foot were subjected to repeated external loading, followed by OCTA–OCE imaging. The aim was to evaluate the feasibility of combined OCTA–OCE imaging for assessing microvascular parameters and tissue elasticity in healthy foot tissue following repeated mechanical loading.

## 2. Materials and Methods

### 2.1. OCT Imaging System

The OCT system used in this study was previously described [33]. The system was developed in our laboratory and uses a non-invasive swept-source OCT (SS-OCT) system with a miniature scanning probe.

**Figure 1:**
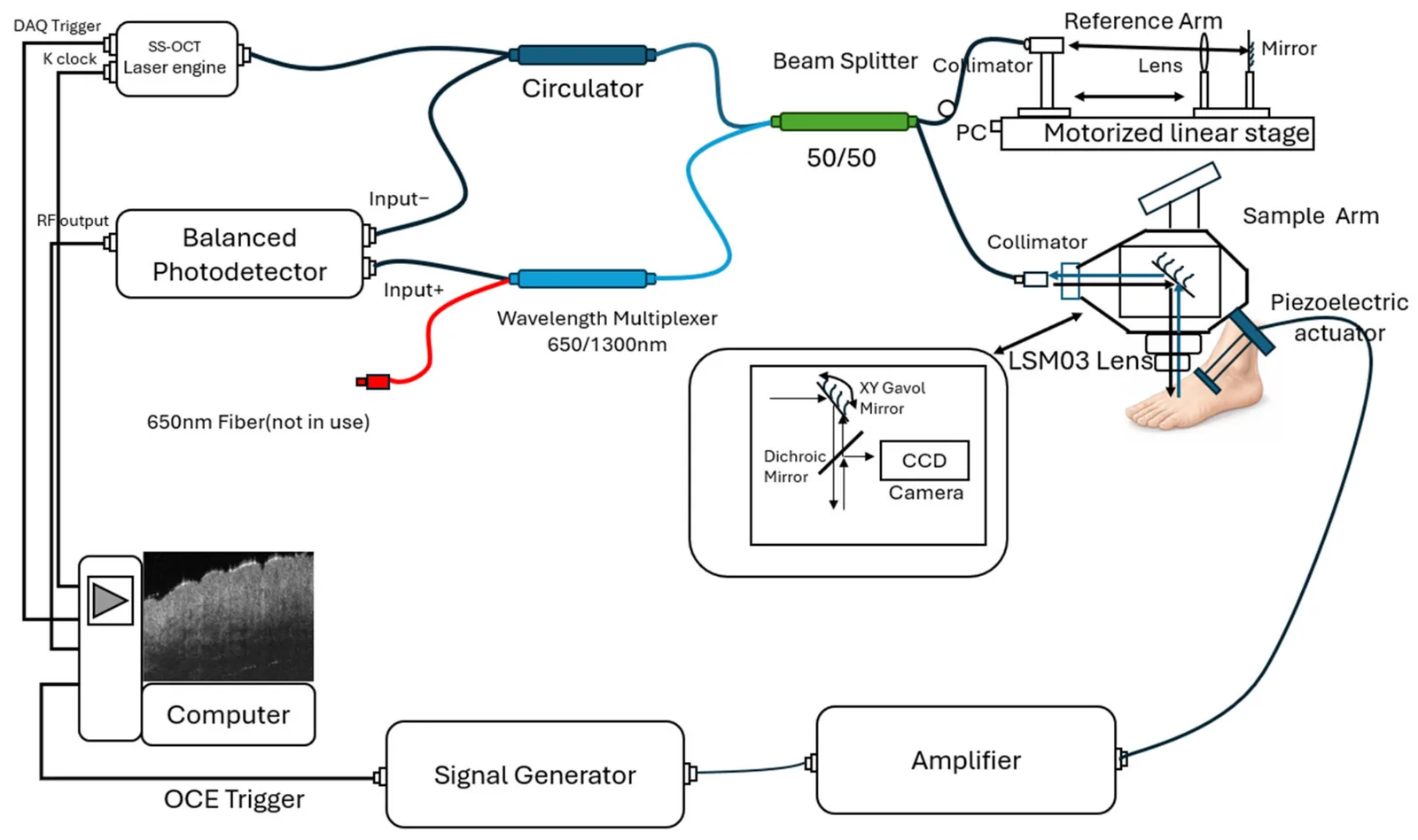
Schematic of the SS-OCT system. PC, polarization controller; CCD, charge-coupled device.

The system used a swept-source laser (SL134050, Thorlabs, Newton, MA, USA) with a center wavelength of 1300 nm and a 100 nm wavelength scan range at −10 dB. The scanning probe supported structural image acquisition, as well as OCE and OCTA data collection, utilizing an objective lens (LSM03, Thorlabs, Newton, MA, USA). A schematic of the system is shown in Figure 1.

For OCE excitation, the piezoelectric actuator was driven by a signal generator (Model 33220A, Keysight Technologies, Inc., Santa Rosa, CA, USA). A 4 kHz square wave with a peak-to-peak voltage of 10 V and a duty cycle of 60% was used to generate mechanical waves on the sample surface. The signal was amplified by a power amplifier (QD-4240, Inter-M Corporation, Yangju-si, Gyeonggi-do, Republic of Korea) with a voltage gain of 4. The SS-OCT system externally triggered the signal generator to synchronize mechanical excitation with OCT acquisition. The scanning protocol followed that described in our previous study [33,34]. At each scanning position, 400 A-lines were repeatedly acquired at the same location to form one M-scan. Each B-scan consisted of 50 M-scans along the X axis. The Y scanning axis then moved the beam across 50 positions to generate an OCE volume of 384 × 50 × 50 × 400 Z×X × Y × M The OCE signal acquisition followed the protocol described in our previous study [33]. The excitation waveform, driving voltage, stimulation duration, acquisition window, and signal processing parameters were kept the same as those used previously. Surface wave propagation was recorded by the OCT system at the marked skin region, and elasticity was estimated using the same reconstruction method. The phase stability of the system was evaluated using a stationary silicone phantom without mechanical excitation. Across three independent measurements, the mean of the median inter-A-line phase noise was 46.5 ± 7.2 milliradian (mrad), corresponding to an axial displacement sensitivity of approximately 4.8 nm.

For OCTA acquisition, the fast-scanning axis performed repeated raster scans along the *X* direction. At each lateral position, 8 repeated B-scan numbers (*N* = 8) were acquired to extract angiographic information from the same location. The galvo then scanned row by row along the *Y* direction to form a 3D data set. The resulting OCTA volume had dimensions of 384 × 400 × 400 × 8 pixels (Z×X × Y × N).

The field of view was 5.1 mm × 5.1 mm in the lateral directions (*X × Y*), with a penetration depth of approximately 1.7 mm in tissue. The total acquisition time was approximately 10 s.

### 2.2. Ethics and Participant Recruitment

This study was approved by the Physical Sciences Ethics Committee (PSEC) of the University of York (approval reference: Zhengshuyi20241125). Ten healthy volunteers were included. Participants had no history of diabetes, peripheral vascular disease, neuropathy, or dermatological conditions affecting the feet. The participants were aged 20–30 years and included 8 males and 2 females. Written informed consent was obtained from all participants before data acquisition.

Each participant underwent OCT imaging at two locations on the right foot: the dorsal and plantar regions. Each region was marked using a sterile surgical pen to ensure consistent imaging at the same site. Four volumes were acquired from each region: one baseline volume before external loading and three volumes acquired after loading following the first, second, and third loading cycles (C1, C2, and C3). Eight volumes were acquired per participant, giving a total of 80 volumes.

### 2.3. Repeated Mechanical Loading Protocol

**Figure 2:**
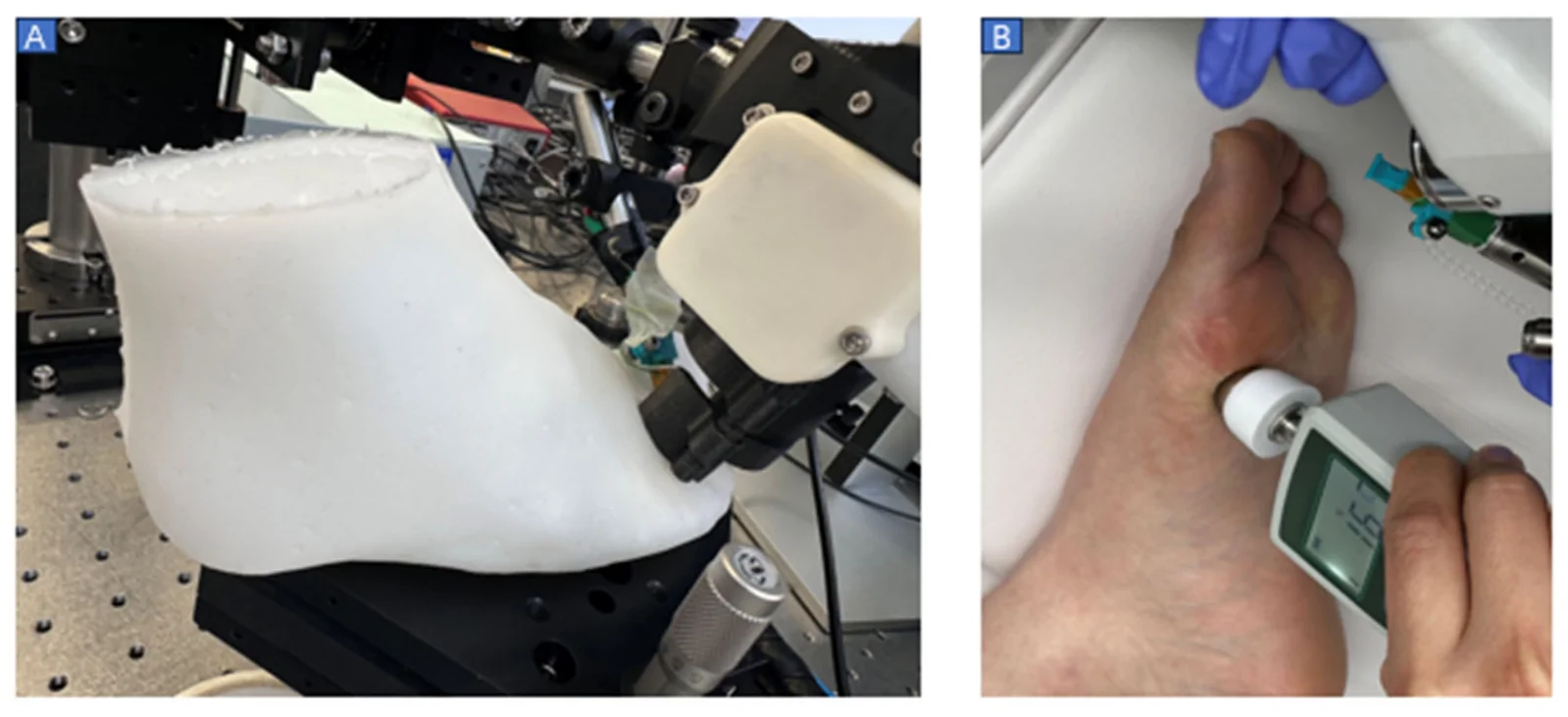
(**A**) OCTA–OCE integrated probe imaging a silicone foot phantom; (**B**) repeated mechanical loading procedure using a handheld force gauge (push–pull dynamometer; measurement range, 0–300 N; resolution, 0.01 N).

The aperture of the contact window was optimized to improve scanning stability [35]. Before in vivo measurements, a silicone foot phantom was fabricated using Ecoflex™ 00-30 silicone (Smooth-On, Inc., Macungie, PA, USA) according to the manufacturer’s instructions. The foot phantom had approximate dimensions of 155 mm × 62 mm × 74 mm (length × width × height). The local thickness at the imaging region was approximately 24 mm. A small amount of white pigment was added to the silicone during preparation to improve optical scattering for OCT imaging. Three repeated OCE measurements were performed on the phantom to evaluate mechanical wave propagation and measurement repeatability before in vivo foot imaging. The experimental setup and loading procedure are shown in Figure 2.

OCT scanning was then performed on the participants. It was performed at four measurement stages for each participant: one baseline acquisition without additional loading, followed by three repeated loading cycles (C1, C2, and C3). A handheld force gauge was used to apply external loading to the marked skin area. For each cycle, the same force was applied for 30 s. The force level was adjusted between participants according to individual tolerance, ranging from 19 to 32 N, with an average of approximately 25 N across the experiment. After each loading cycle, the force was released, and OCTA–OCE imaging started approximately 3 s later. This generated one baseline dataset and three datasets acquired following repeated mechanical loading.

### 2.4. Data Processing and Quantification

An automatic depth-of-interest (DOI) selection algorithm was used to select the analysis layer [36]. Following DOI selection, OCTA projection images were generated using maximum intensity projection. Vessel regions were identified using Otsu’s global thresholding, adaptive thresholding, and Hessian-based vessel enhancement, as described previously [36]. The resulting binary angiography mask (BAM) assigned vessel pixels a value of 1 and background pixels a value of 0. The binary vessel skeleton (BVS) was then generated from the BAM by skeletonization, reducing each vessel to a one-pixel-wide centerline while preserving the vascular network structure. Pixels with a value of 1 in the BVS were defined as skeleton pixels. The equations used to calculate several vascular parameters, including vessel area density (VAD), vessel skeleton density (VSD), vessel diameter index (VDI), vessel tortuosity index (VTI), and the weighted VTI (WTI), are shown below:

(1) $$VAD=\frac{\sum_{n=1}^{n}\sum_{m=1}^{m\;}{\;V}_{\left(x_{n,}y_{m}\right)}}{n\times m}\times 100\%\;$$

Vessel area density (VAD) was defined as the ratio of vessel pixels to the total number of pixels in the analyzed image [37]. $V_{\left(x_{n,}y_{m}\right)}\;$represents the binary value at pixel position $\left(x_{n,}y_{m}\right)$, where a value of 1 represents a vessel pixel and a value of 0 represents a background pixel. The total number of pixels in the image is n×m.

Vessel skeleton density (VSD) was defined as the ratio of vessel skeleton pixels to the total number of pixels in the analyzed image [36]. $S_{\left(x_{n,}y_{m}\right)}$ represents the binary value at pixel position $\left(x_{n,}y_{m}\right)$ in the binary vessel skeleton, where a value of 1 represents a skeleton pixel and a value of 0 represents a background pixel, and n×m represents the total number of pixels in the image.

(2) $$VSD=\frac{\sum_{n=1}^{n}\sum_{m=1}^{m}S_{\left(x_{n,}y_{m}\right)}}{n\times m}\times 100\%\;$$

Vessel diameter index (VDI) was used to estimate vessel diameter from the binary vascular image [38]. $S_{\left(x_{n,}y_{m}\right)}$ represents the skeletonized binary vascular image at pixel position $\left(x_{n,}y_{m}\right)$, and $I_{\left(u,v\right)}$ represents the inverted binary vascular image, where (u,v) denotes pixel coordinates in the inverted image. For each vessel skeleton pixel, the minimum Euclidean distance to the corresponding pixel in the inverted binary image was determined and multiplied by two to estimate the local vessel diameter. The unit for VDI is pixels, with the vascular diameter converted from pixel width to micrometers.

(3) $$VDI=\frac{\sum_{n=1}^{n}\sum_{m=1}^{m}{(s}_{\left(x_{n,}y_{m}\right)}\times 2\times \underset{\left(u,v\right)}{\min}I_{\left(u,v\right)}\sqrt{{{(x}_{n}-u)}^{2}+{{(y}_{m}-v)}^{2}})}{\sum_{n=1}^{n}\sum_{m=1}^{m}{(s}_{\left(x_{n,}y_{m}\right)})}$$

Vessel tortuosity index (VTI) was used to quantify vessel tortuosity [39]. It was calculated from the ratio between the vessel path length L and the straight distance D between the two endpoints of the vessel skeleton.

(4) $$VTI=\left(\frac{L}{D}-1\right)\times 100$$

Weighted vessel tortuosity index (WTI) was calculated by weighting the VTI of each vessel by its VDI. ${VDI}_{a}$ selects the α-th vessel from a total number of A vessels for weighted calculation of VTI [36].

(5) $$WTI=\frac{\sum_{a=1}^{A}{VDI}_{a}\times VTI}{\sum_{a=1}^{A}{VDI}_{a}}$$

**Figure 3:**
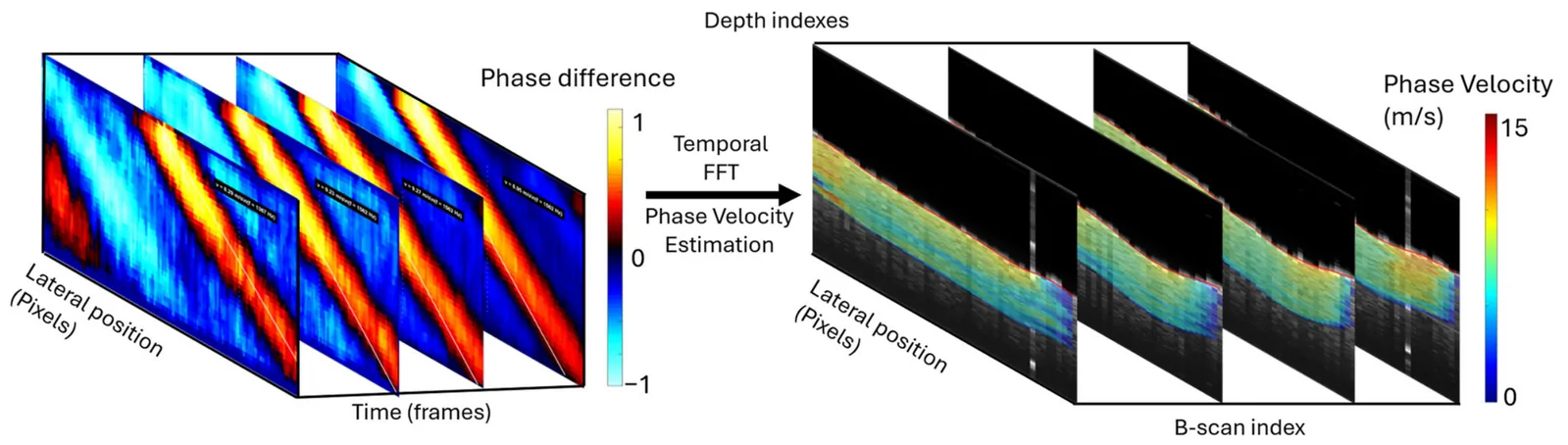
Phase analysis for surface acoustic wave velocity estimation.

The OCE processing workflow for surface acoustic wave (SAW) velocity estimation is illustrated in Figure 3. The phase difference images show the propagation of the mechanical wave over successive time frames at different depths and lateral positions. Fourier transform (FFT) was applied along the temporal dimension to extract the complex signal at the excitation frequency. The wrapped spatial phase, $\phi_{w}\left(x\right)$, was obtained from the argument of the complex FFT signal. The wrapped phase is expressed as:

(6) $$\phi_{w}\left(x\right)=∠\left[Ae^{i\left(k_{rad}x-\omega t+\phi_{0}\right)}\right]$$

where A is the signal amplitude, $k_{rad}$ is the spatial wave number, ω is the angular frequency, and $\phi_{0}$ is the initial phase. The phase returned by the argument operation is wrapped to the principal interval (−π,π]. Phase wrapping was corrected along the lateral wave-propagation direction using one-dimensional phase unwrapping. The unwrapped phase is expressed as:

(7) $$\phi_{u}\left(x\right)=\phi_{w}\left(x\right)+2\pi n$$

where n is an integer selected during the unwrapping process to remove artificial 2π phase discontinuities between adjacent lateral positions and recover a continuous spatial phase profile. The spatial phase gradient was then calculated from the unwrapped phase profile and used to estimate the SAW velocity according to Equation (8). This process was applied across the imaging region to generate a depth-resolved SAW velocity map, showing the spatial distribution of wave velocity within the measured tissue.

The surface acoustic wave (SAW) velocity was estimated using a phase-based method. Temporal phase information was extracted using a Fourier transform, and the spatial phase variation along the wave propagation direction was calculated. The SAW velocity was then determined from the phase gradient [40,41]. ω is the angular frequency,∆x is the propagation distance, and ∆ϕ is the phase delay.

(8) $$C_{saw}\left(\omega \right)=\frac{\omega ∆x}{∆\phi }\;$$

According to the formula for calculating Young’s modulus and surface wave velocity, $C_{saw}$ denotes the surface acoustic wave propagation velocity; ν is Poisson’s ratio; E is Young’s modulus; and ρ is the tissue density [42].

(9) $$C_{saw}=\frac{0.87+1.12\nu }{1+\nu }\sqrt{\frac{E}{2\rho \left(1+\nu \right)}}$$

Young’s modulus was estimated from the measured SAW velocity using Equation (9). The calculation assumes that the measured region can be approximated as a locally homogeneous, isotropic, and nearly incompressible material. For the phantom measurements, the silicone phantom was treated as a homogeneous and isotropic elastic material, with a Poisson’s ratio of 0.5 and a density of 1000 kg/m^3^, following previous surface wave OCE studies [42,43,44]. For skin measurements, a Poisson’s ratio of 0.45 and a density of 1060 kg/m^3^ were used, consistent with previous SAW-OCE measurements of human skin [45]. OCE data quality was assessed after acquisition using two-dimensional cross-sectional images. The surface wave signal was visually inspected based on its signal strength and continuity within the analyzed region. Measurements were considered acceptable when a clear and continuous wave signal could be identified across the imaging region. Acquisitions with weak, discontinuous, or unclear wave signals were considered unreliable and were excluded from further analysis.

All data were acquired using a customized LabVIEW interface (version 2016, National Instruments, Austin, TX, USA) and stored on the laboratory PC for subsequent processing in MATLAB (R2019b, The MathWorks, Inc., Natick, MA, USA).

### 2.5. Statistical Analysis

Statistical analyses were performed using MATLAB (R2019b, The MathWorks, Inc., Natick, MA, USA). Data are presented as mean ± standard deviation. Normality was assessed using the Shapiro–Wilk test for each parameter within each anatomical region and loading condition. A two-way repeated-measures ANOVA was used to evaluate the effects of anatomical region, loading condition, and their interaction. The assumption of sphericity was assessed using Mauchly’s test and was met for all analyzed parameters. For parameters showing a significant main effect of loading in the repeated-measures ANOVA, Bonferroni-adjusted post hoc pairwise comparisons were performed among baseline and the three loading cycles (C1, C2, and C3). Friedman tests were additionally performed separately for the dorsal and plantar regions as non-parametric sensitivity analyses. A *p* value < 0.05 was considered statistically significant.

## 3. Results

### 3.1. OCE Measurements in the Silicone Foot Phantom

Three repeated OCE measurements were performed on the silicone foot phantom to assess measurement repeatability. The mean Young’s modulus within the central imaging region was 150.2 ± 19.1 kPa. Based on the standard deviation across the three repeated measurements, the estimated 95% coefficient of repeatability (CoR) was 52.9 kPa.

### 3.2. Vascular and Elasticity Results for Foot Data

**Figure 4:**
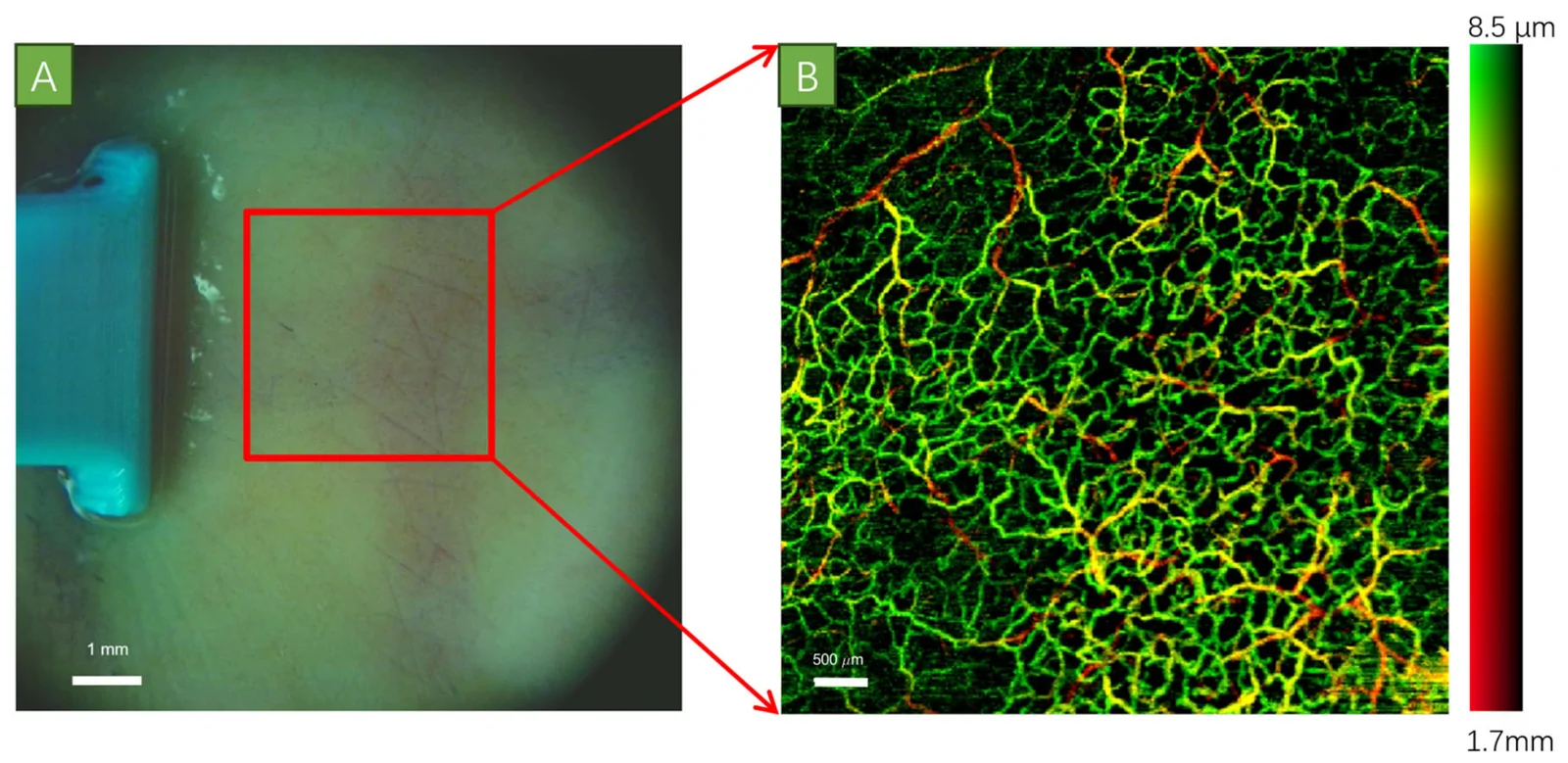
Representative results from the dorsal foot of a healthy participant. (**A**) CCD camera image showing the marked imaging region; (**B**) OCTA *en face* maximum intensity projection. The color bar represents the axial depth of the OCTA signal from 8.5 µm to 1.7 mm and does not indicate that individual vessels extend across the entire depth range.

The representative results from the dorsal foot of one healthy participant are shown in Figure 4. Figure 4A shows the dorsal foot surface captured by the CCD camera. The marked region indicates the imaging location used for repeated measurements, and the red box denotes the acquisition area. Figure 4B presents the OCTA *en* face maximum intensity projection image.

**Figure 5:**
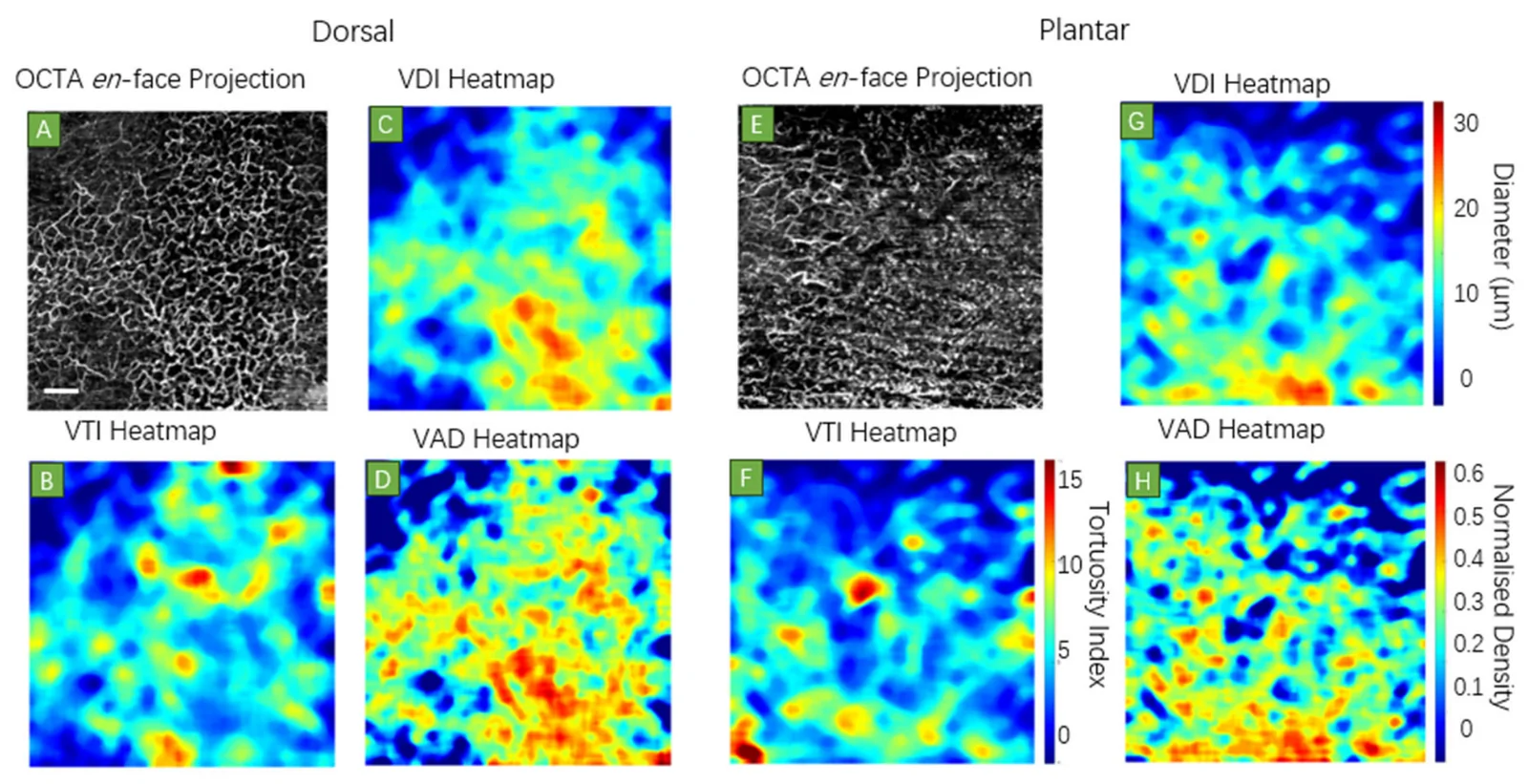
Quantitative assessment of microvascular networks in the dorsal and plantar regions. (**A**,**E**) OCTA *en face* projection images; (**B**,**F**) vessel tortuosity index (VTI) heatmaps; (**C**,**G**) vessel diameter index (VDI) heatmaps; (**D**,**H**) vessel area density (VAD) heatmaps. Scale bars: 500 µm. For each metric, the same color scale and numerical color bar range were used for the dorsal and plantar heatmaps to allow direct comparison between the two regions.

The DOI algorithm was used for quantitative analysis of the foot microvasculature. Representative results are shown in Figure 5. Figure 5A,E show OCTA *en face* projection images of the dorsal and plantar regions, respectively. Figure 5B,F show the corresponding vessel tortuosity index (VTI) heatmaps. Figure 5C,G show vessel diameter index (VDI) heatmaps, while Figure 5D,H show vessel area density (VAD) heatmaps.

Quantitative assessments of the microvascular parameters and Young’s modulus at the dorsal and plantar aspects of the foot are presented in Table 1.

Table 1 summarizes the baseline microvascular parameters and Young’s modulus measured in the dorsal and plantar regions.

As shown in Table 2, the mean microvascular and mechanical parameters are presented for the dorsal and plantar regions at baseline and following each of the three repeated loading cycles. VSD, VAD, VDI, and Young’s modulus are reported for each condition to show the changes across repeated loading.

Relative to baseline, the dorsal VSD was 2.4%, 12.2%, and 9.2% higher following C1, C2, and C3, respectively, while the corresponding changes in VAD were 6.8%, 16.3%, and 13.9%. In the plantar region, VSD was 25.0%, 51.1%, and 61.4% higher than baseline; VAD was 3.8%, 19.0%, and 35.5% higher than baseline following C1, C2, and C3, respectively. These percentage changes are descriptive and were calculated from the group mean values. VDI showed smaller changes. The dorsal VDI was 3.6%, 3.9%, and 3.8% higher than baseline following C1, C2, and C3, respectively. The corresponding changes in the plantar region were 3.0%, 4.5%, and 6.1%.

Young’s modulus was higher than baseline following repeated loading. In the dorsal region, Young’s modulus was 22.9%, 32.3%, and 39.0% higher following C1, C2, and C3, respectively. The corresponding values in the plantar region were 18.1%, 29.2%, and 39.0% higher than baseline.

To assess whether these numerical trends were statistically significant, Friedman tests were performed across the four conditions, as summarized in Table 3.

Within each foot region, Friedman tests were used to assess the overall differences among baseline and the three repeated loading cycles. Significant overall differences were observed for plantar VSD (*p* = 0.006) and Young’s modulus in both the dorsal and plantar regions (*p* < 0.001). To examine the effects of anatomical region and loading condition across both foot regions, repeated-measures ANOVA was also performed. This analysis assessed the effects of region, loading condition, and their interaction, as summarized in Table 4.

Repeated-measures ANOVA was performed to evaluate the effects of anatomical region, loading condition, and their interaction. A significant region effect was observed for VSD (F(1, 5) = 13.88, *p* = 0.014). Significant loading effects were found for VSD (F(3, 15) = 7.07, *p* = 0.003), VAD (F(3, 15) = 8.37, *p* = 0.002), and Young’s modulus (F(3, 18) = 117.97, *p* < 0.001). No significant region × loading interaction was observed for any parameter (all *p* > 0.05).

For parameters showing a significant loading effect in the repeated-measures ANOVA, Bonferroni-adjusted post hoc pairwise comparisons were performed among baseline and the three loading cycles (Table 5). For VSD, a significant difference was observed between C1 and C3 (adjusted *p* = 0.046). For VAD, significant differences were observed between baseline and C3 (adjusted *p* = 0.048) and between C1 and C3 (adjusted *p* = 0.030). For Young’s modulus, significant differences were observed among all pairwise loading-condition comparisons (all adjusted *p* < 0.05). These results indicate that Young’s modulus showed the most consistent progressive change across repeated loading, whereas pairwise differences in the vascular parameters were less consistent.

## 4. Discussion

This study evaluated the feasibility of combined OCTA–OCE imaging for assessing microvascular and mechanical responses in healthy foot tissue following repeated loading. The Friedman analysis showed significant differences in plantar VSD and Young’s modulus in both regions. The repeated-measures ANOVA identified significant loading effects for VSD, VAD, and Young’s modulus. A significant region effect was also observed for VSD. These findings support the potential of combined OCTA–OCE for assessing vascular and mechanical responses following repeated loading in healthy foot tissue.

Previous study has shown that sustained mechanical pressure can reduce microvascular perfusion and may be followed by reactive hyperemia after pressure release [46]. The plantar region is regularly exposed to mechanical loading and may be particularly relevant for assessing changes in microvascular function [5]. In the present study, the repeated-measures analysis also showed a significant overall loading effect for VAD. However, the Friedman analyses within the dorsal and plantar regions did not show significant differences for VAD. As imaging was performed after force release, this finding may reflect changes in the microvascular state following mechanical loading rather than the vascular response during compression. Previous studies have also reported changes in vessel density and vessel diameter associated with altered foot microcirculation [29,30,47]. The vascular measurements obtained in this study were broadly within the ranges reported in previous studies. Combined assessment of microvascular and mechanical parameters may, therefore, provide additional information on tissue response to loading and may have potential for future assessment of tissue vulnerability in the diabetic foot [48].

In addition to the vascular parameters, Young’s modulus was higher following repeated loading. Relative to baseline, the dorsal Young’s modulus was 22.9%, 32.3%, and 39.0% higher following C1, C2, and C3, respectively. The plantar region showed a similar pattern, with a Young’s modulus 18.1%, 29.2%, and 39.0% higher than baseline. The Friedman test showed significant overall differences in Young’s modulus in both regions (*p* < 0.001), and the repeated-measures ANOVA identified a significant loading effect. These findings indicate higher measured tissue stiffness following repeated mechanical loading [12,49,50,51].

From a biological perspective, the vascular changes observed in this study may be related to microcirculatory responses following external mechanical loading. Mechanical loading can temporarily compress superficial vessels, while reperfusion may occur after force release [31]. This may contribute to changes in perfusion-related parameters measured shortly after loading. In the plantar region, VSD showed a significant overall difference between loading conditions, with values higher than baseline following repeated loading. VDI showed smaller numerical changes following repeated loading, suggesting that different vascular parameters may respond differently during the early recovery period [46,52]. The higher Young’s modulus observed following repeated loading may reflect a short-term mechanical response of the tissue. Skin is a nonlinear and viscoelastic material, and its mechanical properties can change with loading and deformation [53]. Tissue structure may also influence the measured mechanical response. However, the present study did not directly investigate the underlying mechanism. From a clinical perspective, combined OCTA and OCE may provide complementary information to existing diabetic foot assessment methods. Monofilament testing mainly evaluates protective sensation, Doppler ultrasound assesses larger vessel blood flow, and plantar pressure mapping measures external pressure distribution [9,54]. In contrast, OCTA and OCE can assess local microvascular and mechanical properties at the same imaging site. This approach may, therefore, provide additional high-resolution information for assessing foot tissue status [47,48].

This study has several limitations. First, the applied force was adjusted according to participant tolerance and varied between subjects. This limited the assessment of the relationship between force magnitude and the measured vascular and mechanical responses. Variation in tissue deformation may also have influenced surface wave propagation and the OCE-derived Young’s modulus [55]. Second, the sample size was small, and the study included only young healthy participants. This limits the generalizability of the findings and may reduce the ability to detect smaller vascular changes. Larger studies with a wider range of participants are needed to confirm the observed responses and to establish more reliable estimates of effect size. Third, OCTA and OCE measurements were initiated approximately 3 s after force release. Therefore, the measurements reflect an early post-loading response rather than the tissue response during loading. The recovery interval was not systematically varied, and possible order or carry-over effects between repeated loading cycles cannot be excluded. Although Young’s modulus increased progressively and showed significant pairwise differences between loading conditions, the vascular parameters showed less consistent pairwise differences. Therefore, the observed changes cannot be conclusively attributed to cumulative loading effects. The relatively small sample size may also have limited the statistical power to detect smaller vascular changes. Future studies should include controlled recovery intervals to distinguish cumulative loading effects from order-related and recovery effects. Further work is also needed to determine whether similar vascular and mechanical responses occur in populations with impaired microcirculation or altered tissue mechanics, such as individuals with diabetes.

Age, sex, and physical activity may influence microvascular regulation and tissue mechanical properties. Older adults may show different vascular recovery and skin stiffness compared with younger adults [56]. Sex may also affect skin structure, blood perfusion, and elasticity because of differences in skin thickness and tissue composition [57]. Physical activity and repeated plantar loading may influence foot microcirculation and tissue structure [58]. Future studies should include older adults, more balanced male and female groups, and participants with diabetes. This would improve the generalizability of the findings. Collecting OCTA–OCE features from these populations may also support future machine learning analysis. Differences in foot shape, tissue structure, and loading may influence OCTA and OCE results. OCTA signals depend on detectable blood flow and do not directly measure absolute vascular density. Probe position may also introduce measurement variation.

## 5. Conclusions

This study demonstrates the feasibility of combined OCTA–OCE imaging for assessing microvascular and mechanical responses in healthy foot skin following repeated loading. Significant changes were observed in plantar VSD and Young’s modulus. The overall analysis also identified loading effects for VSD, VAD, and Young’s modulus. Future studies should include older adults, more balanced male and female groups, and participants with diabetes to further evaluate the potential of this method for assessing changes following loading in the diabetic foot.

## Figures and Tables

**Table 1:** Baseline microvascular and elasticity measurements in the dorsal and plantar regions of healthy participants.

Region	VSD (%)	VAD (%)	VDI (µm)	WTI	Young’s Modulus (kPa)
Dorsal	5.31 ± 1.60	16.97 ± 5.71	35.97 ± 1.50	15.72 ± 1.36	248.93 ± 20.25
Plantar	2.80 ± 0.38	12.75 ± 4.07	39.67 ± 3.97	17.26 ± 4.10	250.23 ± 22.24

Note: VSD, vessel skeleton density; VAD, vessel area density; VDI, vessel diameter index; WTI, weighted vessel tortuosity index. Values are presented as mean ± standard deviation (SD).

**Table 2:** Quantitative OCTA and OCE parameters in the dorsal and plantar regions at baseline and following repeated loading.

Region	Dorsal				Plantar			
Metrics	Baseline	C1	C2	C3	Baseline	C1	C2	C3
VSD (%)	5.31 ± 1.60	5.44 ± 1.43	5.96 ± 1.07	5.80 ± 1.17	2.80 ± 0.38	3.50 ± 0.48	4.23 ± 0.95	4.52 ± 1.15
VAD (%)	16.97 ± 5.71	18.13 ± 5.79	19.73 ± 4.79	19.33 ± 4.98	12.75 ± 4.07	13.23 ± 1.79	15.17 ± 4.85	17.27 ± 3.90
VDI (μm)	35.97 ± 1.50	37.25 ± 2.14	37.39 ± 2.10	37.32 ± 2.24	39.67 ± 3.97	40.85 ± 5.94	41.44 ± 4.27	42.08 ± 3.47
Young’s modulus (kPa)	248.93 ± 20.25	306.05 ± 21.99	329.38 ± 14.11	346.10 ± 26.16	250.23 ± 22.24	295.45 ± 17.91	323.27 ± 17.01	347.87 ± 18.26

Note: Values are presented as mean ± standard deviation (SD). C1, first loading cycle; C2, second loading cycle; C3, third loading cycle.

**Table 3:** Statistical analysis of loading-related changes in vascular and mechanical parameters.

Region	Parameter	Friedman Test *p* Value
Dorsal	VSD	0.48
	VAD	0.45
	VDI	0.16
	Young’s modulus	**2.75 × 10 ^−5^**
Plantar	VSD	**6.3 × 10 ^−5^**
	VAD	0.08
	VDI	0.26
	Young’s modulus	**2.75 × 10 ^−5^**

Note: Statistical significance was defined as *p* < 0.05. Significant values are shown in bold. Friedman tests were used as nonparametric sensitivity analyses within each region.

**Table 4:** Repeated-measures ANOVA for the effects of anatomical region and loading condition.

Parameter	Region Effect *p* Value	Loading Effect *p* Value	Region × Loading Interaction *p* Value
VSD	**F(1, 5) = 13.88, *p* = 0.014**	**F(3, 15) = 7.07, *p* = 0.003**	F(3, 15) = 0.93, *p* = 0.449
VAD	F(1, 5) = 2.22, *p* = 0.196	**F(3, 15) = 8.37, *p* = 0.002**	F(3, 15) = 0.29, *p* = 0.833
VDI	F(1, 5) = 6.33, *p* = 0.053	F(3, 15) = 2.10, *p* = 0.144	F(3, 15) = 0.35, *p* = 0.787
Young’s modulus	F(1, 6) = 0.26, *p* = 0.629	**F(3, 18) = 117.97, *p* < 0.001**	F(3, 18) = 1.70, *p* = 0.203

Note: F values, degrees of freedom, and *p* values are reported. Statistical significance was defined as *p* < 0.05. Significant results are shown in bold. Sphericity was assessed before analysis. Only participants with complete paired data across both anatomical regions and all loading conditions were included in the repeated-measures ANOVA; therefore, the number of participants included varied between parameters.

**Table 5:** Bonferroni-adjusted pairwise comparisons following significant loading effects in the repeated-measures ANOVA.

Parameter	B vs. C1	B vs. C2	B vs. C3	C1 vs. C2	C1 vs. C3	C2 vs. C3
VSD	1.00	0.23	0.16	0.25	**0.046**	1.00
VAD	1.00	0.29	**0.048**	0.70	**0.03**	0.64
Young’s modulus	**<0.001**	**<0.001**	**<0.001**	**0.013**	**0.004**	**0.028**

Note: Values are Bonferroni-adjusted *p* values from post hoc pairwise comparisons. Significant differences (adjusted *p* < 0.05) are shown in bold. B, baseline; C1–C3, loading cycles 1–3.

## Data Availability

The data presented in this study are available on request from the corresponding author. The data are not publicly available due to ethical restrictions.
